# EdgeSugarcane: a lightweight high-precision method for real-time sugarcane node detection in edge computing environments

**DOI:** 10.3389/fpls.2025.1626725

**Published:** 2025-07-07

**Authors:** Zhenhui Zheng, Lijiao Wei, Kangmin Lin, Weihua Huang, Shuo Wang, Dongjie Du, Tao Wu

**Affiliations:** ^1^ College of Engineering, South China Agricultural University, Guangzhou, China; ^2^ Agricultural Machinery Research Institute, Chinese Academy of Tropical Agricultural Sciences, Zhanjiang, Guangdong, China; ^3^ Key Laboratory of Agricultural Equipment for Tropical Crops, Ministry of Agriculture and Rural Affairs, Zhanjiang, Guangdong, China

**Keywords:** sugarcane, YOLOv8, TensorRT, edge computing, lightweight

## Abstract

Accurate detection of sugarcane nodes in natural environments is crucial for realizing intelligent sugarcane cutting and precise planting localization. However, current sugarcane node detection models often face challenges such as large parameter sizes, poor adaptability to deployment environments, and limited real-world detection accuracy. To address these challenges, this research proposes a high-precision and lightweight EdgeSugarcane detection model. Firstly, based on YOLOv8, an improved EdgeSugarcane model is proposed. By introducing an interactive attention mechanism across channel and spatial dimensions, the model’s ability to represent node features is enhanced. Then, combined with TensorRT acceleration and optimization, the optimal FP16 quantization deployment scheme is proposed. Finally, end-to-end deployment is implemented on the NVIDIA Orin NX edge device, and its performance and resource consumption in practical applications are analyzed in depth. The experimental results show that EdgeSugarcane achieves a precision of 0.935, a recall of 0.8, and a mAP of 0.87 on the test set, with a model size of 89.9 MB. Compared to YOLOv8, the mAP is improved by 0.6%, and the inference speed is increased by 44%. With lossless precision, the inference time after FP16 quantization is only 1.9ms, a 3.3-fold improvement compared to before optimization, and the model size changes very little. On the NVIDIA Orin NX device, the single-frame inference, pre-processing, and post-processing times are 1.5ms, 60.6ms, and 4.4ms, respectively. The EdgeSugarcane model demonstrates excellent real-time performance and high accuracy under natural field conditions, offering a viable solution for integration into edge-based robotic systems for intelligent sugarcane cutting and precision planting.

## 1 Introduction

Sugarcane is an important strategic agricultural commodity widely cultivated globally, playing a significant role in people’s lives and national economic development (Karp et al., 2022; Huang et al., 2020; Qian et al., 2024). China is the world’s third-largest sugarcane producer, after Brazil and India, and the low level of harvesting mechanization is one of the key factors affecting the sustainable development of China’s sugarcane industry (Que et al., 2024; Liu et al., 2024; Lu and Wu, 2023). Automation of sugarcane node cutting and harvesting is a crucial part of achieving sugarcane mechanization. In this study, the focus is specifically on intelligent sugarcane node cutting, where accurate identification of sugarcane nodes enables precise segmentation and planting. Node detection remains a key research topic in this process. The application of sugarcane node recognition technology helps to significantly improve production efficiency, reduce costs, enhance crop monitoring capabilities, and support precision agriculture. Existing sugarcane node detection methods suffer from shortcomings such as low detection accuracy, high model complexity, and less-than-ideal efficiency. Therefore, designing a high-performance, easy-to-deploy, and low-cost sugarcane node detection system is essential for intelligent sugarcane field operations.

Scholars have conducted relevant research on sugarcane node detection systems. Tan et al. (2014) designed a sugarcane node sorting system based on machine vision to automatically sort sugarcane nodes with nodes and sugarcane nodes without nodes. Huang et al. (2017) proposed sugarcane node recognition based on local mean value, which performs threshold segmentation, morphological processing, and maximum area selection on the H component of its HSV color space, achieving a recognition rate of 90.77% with a better combination, and an average time of 0.481539s. Zhou et al. (2020) proposed a new design for a machine vision-based sugarcane node cutting system, with a recognition rate of 93% and an average time of 0.539s. Chen et al. (2021b) proposed a sugarcane node recognition algorithm based on the local pixel sum of the minimum point of the vertical projection function. The dual-node recognition rate was 98.5%, with an average time consumption of 0.21s. Yang et al. (2020) proposed a gradient-based method for sugarcane multi-node identification, achieving an accuracy rate of 96.8952%. Chen et al. (2021) explored sugarcane node detection based on wavelet analysis, detecting 99.63% of sugarcane node samples with an error rate of 0.37% and a response time of 0.25 seconds. Meng et al. (2019) proposed a sugarcane node recognition technology based on wavelet analysis, with a maximum positioning error of less than 2.5 mm and a maximum delay of 0.25 seconds. Despite these methods have achieved some success in sugarcane node detection tasks, there are still some significant shortcomings. The above studies automate the identification of sugarcane node numbers by fusing artificially extracted features and using traditional machine learning algorithms. However, in terms of real-time performance and robustness, traditional methods still have considerable room for improvement, and there is an urgent need for more adaptable new algorithms to cope with complex and ever-changing sugarcane image data.

In recent years, many researchers have gradually devoted themselves to the related fields of sugarcane node detection, especially through deep learning technology to improve detection accuracy and efficiency. Zhou et al. (2022) studied a sugarcane node identification and localization algorithm combining YOLOv3 with traditional computer vision methods to improve recognition rates during automatic cutting. Chen et al. (2021a) conducted field sugarcane node recognition based on deep learning combined with data expansion, achieving an average accuracy of 95.17% and a detection speed of 69f/s. Wang et al. (2022b) proposed a machine vision-based sugarcane node cutting system in seed-front mode, with a recognition rate of no less than 94.3% and an average accuracy of 98.2%. Zheng et al. (2024) developed an efficient sugarcane node detection method based on YOLOv8, with a precision of 0.973, recall of 0.958, and mAP of 0.974. Zhu et al. (2022) explored binocular vision-based sugarcane node spatial localization for harvesting robots using improved YOLOv4, which improved average accuracy. Dai et al (2024b) proposed an improved YOLOv5-based intelligent recognition system for sugarcane joints, achieving a mean average precision (mAP) of 89.89% with a single image detection time of approximately 1.87 seconds. Dai et al (2024a) proposed an intelligent sugarcane node recognition system based on enhanced YOLOv5s, with recognition accuracy, recall, and mAP values reaching 89.89%, 89.95%, and 92.16%, respectively, and a single image inference time of only 22ms. Xie et al. (2024) proposed a cane node detection method based on improved YOLOv5s, with a stem node recognition accuracy rate of 96.4%, a recall rate of 96.8%, and an average precision mean mAP0.5of 98.4%. Chen et al. (2023) adopted YOLOv4-tiny with a network slimming algorithm for sugarcane node identification, effectively reducing model complexity and making it suitable for embedded and mobile devices. Wang et al. (2022b) proposed the use of deep learning for sugarcane node detection and localization, with an average accuracy of 99.11% and a detection accuracy of 97.07%. Hu et al. (2025) proposed an improved YOLOv8n-ghost model, with a real-time detection speed of nearly 30FPS. Xie et al. (2023) proposed a sugarcane node recognition algorithm based on improved YOLOv5, with an average accuracy of 97.6% and a model size of 2.6MB. Although deep learning technology has shown promise in sugarcane node detection, most related research and practices tend to rely on high-performance computing equipment, often requiring significant computing resources, storage space, and high hardware investment. This is significantly different from the needs of actual agricultural operations. Many small-scale farmers have limited affordability in terms of equipment and funding, making it difficult to adopt these technologies widely. Based on the above multi-dimensional analysis and comprehensive consideration, this high-cost deep learning method does not meet the requirements of low cost and high efficiency in practical applications, and it is difficult to be effectively deployed and promoted in widespread agricultural production.

Despite the promising results of deep learning in sugarcane node detection, most existing solutions still suffer from limited generalizability in outdoor environments, large model sizes that hinder deployment, and high computational demands that are impractical for real-world use, especially in resource-constrained farming contexts. In response to these challenges, this paper proposes a lightweight and hardware-adaptive sugarcane node detection method based on an improved YOLOv8 framework. The proposed approach emphasizes accuracy, low latency, and minimal resource consumption, aiming to meet the practical needs of intelligent sugarcane planting on affordable edge devices. The main contributions of this research include: (1) To address the issue of poor feature representation and background interference in complex natural scenes, we embed a Triplet Attention module into the YOLOv8 backbone, enhancing spatial-channel feature interaction and boosting node recognition accuracy. (2) To overcome computational inefficiency and large model sizes that hinder deployment, we employ a TensorRT-based FP16 quantization and acceleration strategy tailored for edge hardware, ensuring low-latency, resource-efficient inference without sacrificing precision. (3) To validate practical feasibility, we implement a full end-to-end deployment on the NVIDIA Orin NX edge platform and verify the model’s robustness through real-environment field tests under varying light and background conditions.

## 2 Materials

### 2.1 Image acquisition

This study was conducted from November 2023 to October 2024 at the sugarcane field (21°10′N, 110°16′E) of the Agricultural Machinery Research Institute, Chinese Academy of Tropical Agricultural Sciences in Zhanjiang City, Guangdong Province, China. The region belongs to the tropical monsoon climate zone, characterized by fertile soil and synchronous water and heat availability, providing a coordinated ecological base for sugarcane growth in terms of light, temperature, water, and soil.

**Figure 1:**
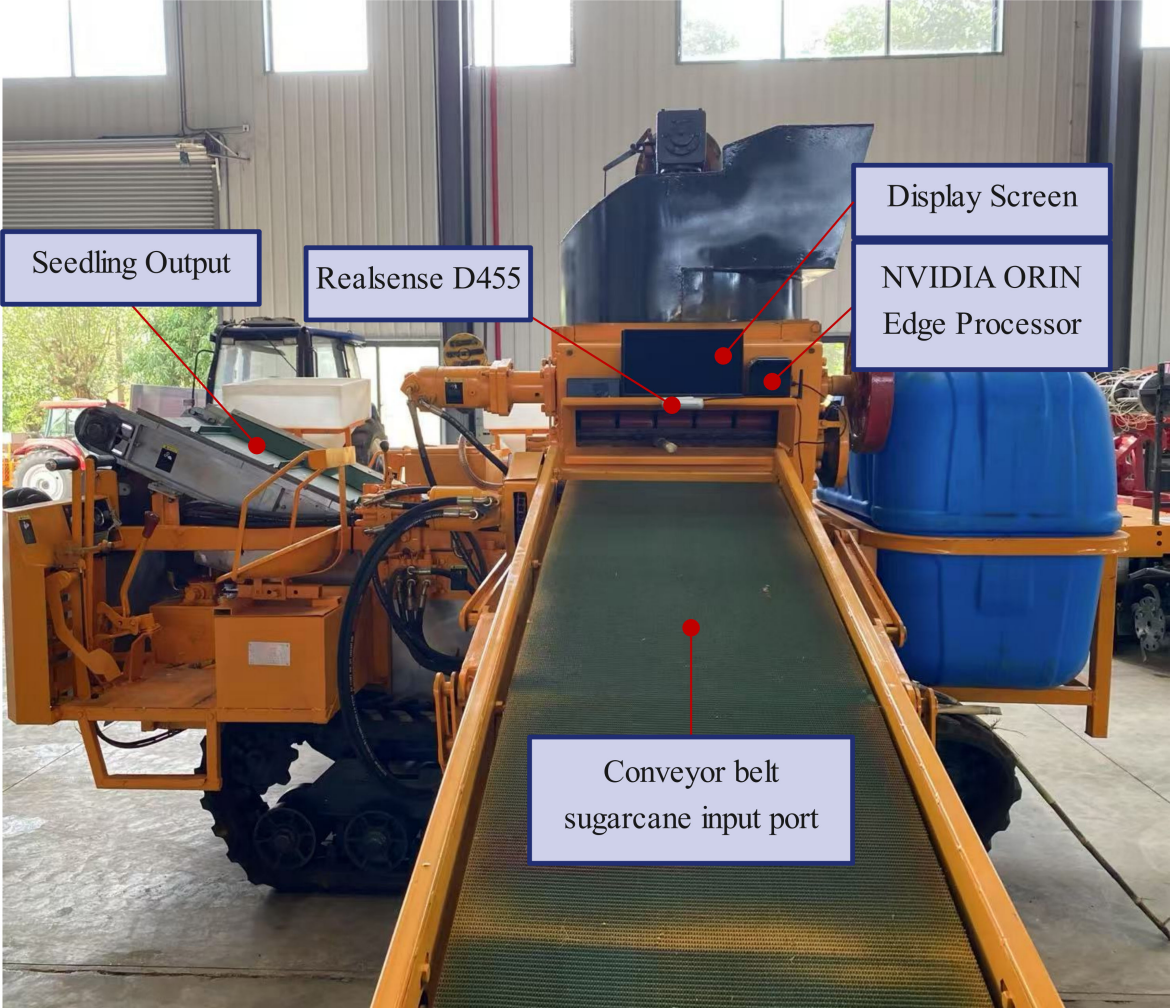
Automated sugarcane node cutting machine.

Based on this foundation, we employed a self-developed automated sugarcane node cutting machine as the primary acquisition device, equipped with a power supply, display screen, NVIDIA ORIN edge processor, and a conveyor belt with an input port, as shown in **Figure 1**. For indoor image acquisition, we used an Apple iPhone 11 and an Intel RealSense D455 camera in a laboratory environment with consistent natural lighting. The iPhone 11 captured RGB images under standard auto settings, with a focal length of 26 mm and an aperture of approximately f/1.8, while the RealSense D455 was operated using its default RGB parameters to ensure reliable spatial consistency and color representation. For outdoor image acquisition, sugarcane in natural field conditions was photographed using Huawei Mate 60 Pro and iPhone 11 devices, also under automatic settings with similar optical parameters (26 mm focal length, ~f/1.8 aperture). This allowed the dataset to capture realistic variations in light, background, and occlusion. In total, 626 high-quality sugarcane images were collected, including 360 from indoor scenes and 266 from outdoor scenes. The inclusion of both controlled and natural conditions ensures diversity in data characteristics, which enhances the model’s generalization capability and robustness. These acquisition conditions are fully replicable, providing a reliable basis for future studies in sugarcane node detection and localization.

### 2.2 Data preprocessing

This study began with the selection and annotation of 600 clear and representative sugarcane images. Labelmg (GitHub Repository. http://github.com/tzutalin/labelImg), a commonly used software in the field of object detection, was employed as the annotation tool. To ensure that the category information and coordinate location of each sugarcane within the images were accurately recorded, each image underwent meticulous labeling. The filtered dataset was divided into training, validation, and testing sets in a 7:1.5:1.5 ratio. Specifically, 350 images from indoor scenes and 250 images from outdoor scenes were allocated to the training, validation, and testing sets according to this ratio, as shown in **Table 1**.

**Table 1:** Details of the data set.

DatasetScence	Training set	Validation set	Testing set
Indoor	245	53	52
Outdoor	175	38	37

## 3 Lightweight sugarcane node detection network

### 3.1 YOLOv8 model

In intelligent planting and harvesting systems in the wild, the accurate identification of sugarcane nodes is crucial for achieving automatic positioning and management. YOLOv8 has emerged as a mature and stable object detection framework, offering an ideal balance of efficiency and accuracy that makes it particularly suitable for intelligent planting and harvesting systems across diverse agricultural scenarios.

The core advantage of YOLOv8 lies in its advanced network architecture design. This model employs a lightweight backbone network and effectively fuses multi-scale feature information through a feature pyramid network (FPN) structure in the neck. Finally, a novel output head is used for object recognition and localization. This architecture allows YOLOv8 to significantly improve computational efficiency while maintaining high detection accuracy. In addition, the deployment cost of YOLOv8 is extremely low, allowing it to run easily on edge computing devices without relying on powerful hardware support. Therefore, YOLOv8 is selected as the basic framework for sugarcane node detection.

However, despite the excellent performance of YOLOv8 under ideal conditions, its detection accuracy is still insufficient in complex natural environments. Furthermore, its model size and computational resource requirements still make it difficult to achieve optimal performance on some edge devices. Therefore, in order to improve the environmental adaptability and lightweight characteristics of the model, we need to improve the existing YOLOv8 model.

### 3.2 EdgeSugarcane model

To address the challenges of occlusion, inter-class differences, and computational resource limitations encountered by YOLOv8 in complex environments, we propose the Triplet Attention module. This module enhances feature fusion and attention mechanisms by considering three information sources simultaneously to overcome these issues. In the EdgeSugarcane network, we introduce this module to achieve a lightweight design and inter-layer collaboration, significantly improving detection accuracy and speed in complex scenarios.

#### 3.2.1 Triplet attention module

**Figure 2:**
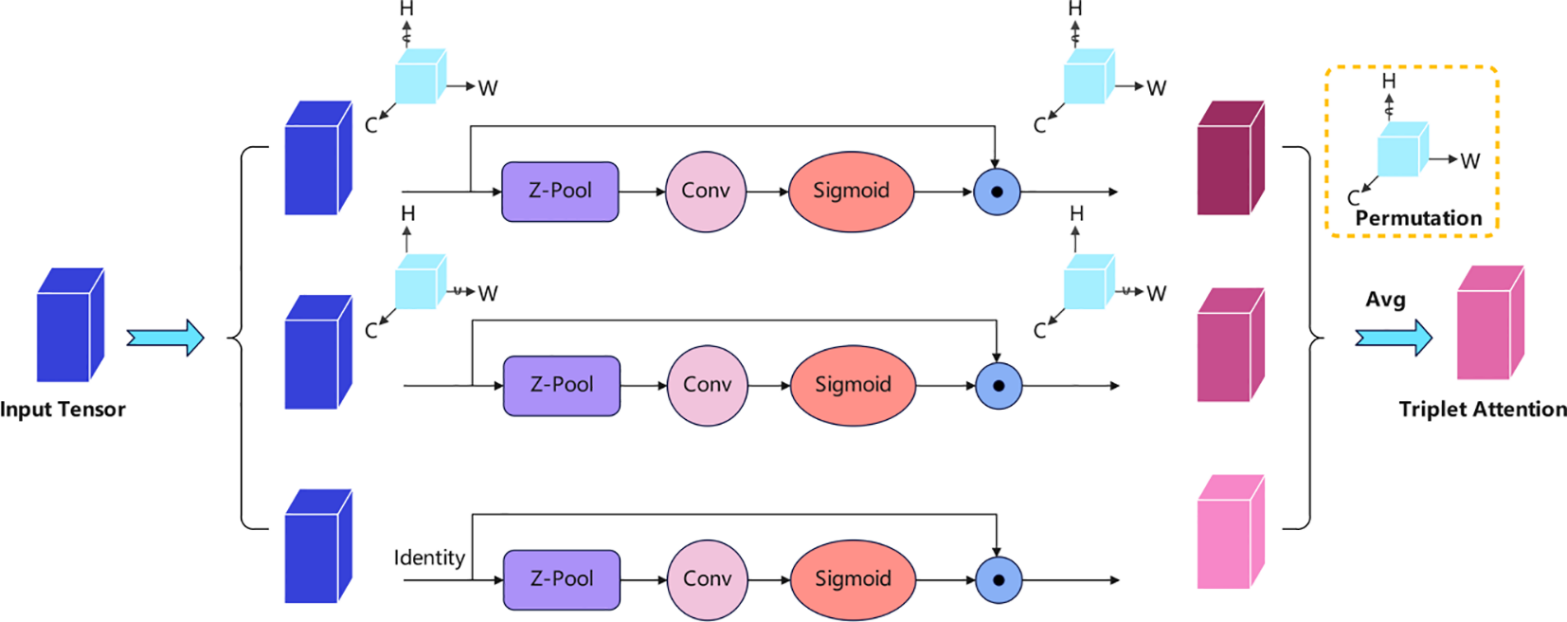
Triplet attention with a three-branch architecture.

Triplet Attention is an attention mechanism module. Compared with commonly used attention mechanisms such as SE (Squeeze-and-Excitation) and CBAM (Convolutional Block Attention Module), Triplet Attention offers enhanced modeling capacity by capturing cross-dimensional interactions among channel, height, and width simultaneously. This property is particularly valuable in the sugarcane node detection task, where small features are often occluded or distorted by lighting and background interference. Therefore, Triplet Attention was selected for its superior ability to refine feature maps in complex natural scenes. It aims to improve feature expression. It does this by capturing dependencies between different dimensions of the input tensor. As shown in **Figure 2**, each branch models cross-dimensional dependencies. These are between pairs of dimensions: Channel and Height (C, H), Channel and Width (C, W), and Height and Width (H, W). This allows for effective information fusion. Specifically, given an input tensor X∈ R C × H × W, the calculation flow of the three branches is as follows:

Pool is responsible for reducing the C dimension of the Tensor to 2 dimensions, specifically by concatenating the average-pooled feature and the max-pooled feature along that dimension. It can be represented by the following equation:


$$\text{Z}-\text{pool}\left(\text{X}\right)=\left[{\text{MaxPool}}_{0\text{d}}\left(\text{X}\right),{\text{AvgPool}}_{0\text{d}}\left(\text{X}\right)\right]$$

The first branch transforms the input tensor and then performs interaction between the H and C dimensions. X is rotated 90° along the H-axis. This results in a tensor ‘ ${\hat{\chi }}_{1}$ ‘ with shape W × H × C. The Z-pool operation is then used to extract features. These features are then passed through a convolution and a Sigmoid activation function to generate the channel attention weights, A_c. The calculation method is as follows;


$$\text{A}\_\text{c}=\text{σ}\left(\text{Conv}\left(\text{Z}\left({\hat{\chi }}_{1}\right)\right)\right)$$

The second branch is similar to the (C, H) branch. It performs interaction between the C and W dimensions. X is rotated 90° along the W-axis. This results in a tensor ‘ ${\hat{\chi }}_{2}$ ‘ with shape H × C × W. This generates the spatial attention weights, A_s. The calculation method is as follows:


$$\text{A}\_\text{s}=\text{σ}\left(\text{Conv}\left(\text{Z}\left({\hat{\chi }}_{2}\right)\right)\right)$$

The third branch directly interacts the H and W dimensions. Features are extracted using the Z-pool operation. These features are then processed by a convolution and a Sigmoid activation function to generate the attention weights, A_hw . The calculation method is as follows:


A_hw=σ(Conv(Z(X)))

Finally, after restoring each rotated branch to its original orientation, the results from the three branches are averaged. This yields the final refined tensor output:


$$\text{Output }=\;\frac{1}{3}\left(\bar{{\hat{\chi }}_{1}\text{ A}\_\text{c}}\;+\bar{{\;\hat{\chi }}_{2}\text{A}\_\text{s}}\;+\text{ X A}\_\text{hw}\right)$$

#### 3.2.2 EdgeSugarcane network architecture

**Figure 3:**
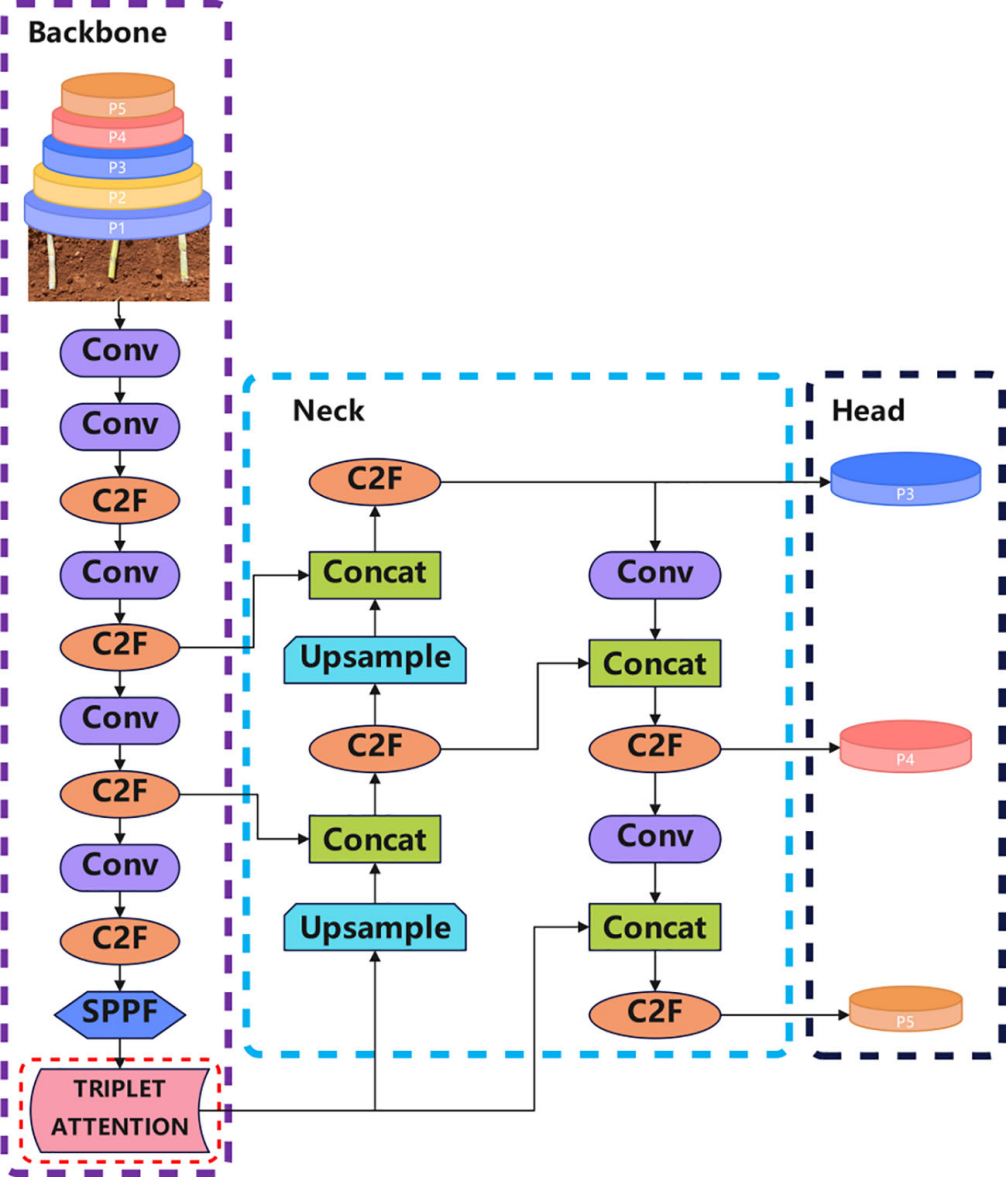
Architecture of EdgeSugarcane network. The improvement is marked by the red dotted box, which is the introduced Triplet Attention Module.

The improved YOLOv8 architecture integrates the Triplet Attention module immediately after the SPPF module, which follows the final C3 block in the backbone, as shown in **Figure 3**. This placement allows the module to act directly on the high-level semantic features output by the backbone before they are passed to the neck for multi-scale fusion. The Triplet Attention mechanism applies cross-dimensional attention weighting across channel and spatial dimensions, effectively enhancing node edge and texture representation while suppressing background noise. This structure not only maintains a lightweight footprint but also significantly boosts detection accuracy for small and densely distributed targets under occlusion or uneven lighting. The optimized feature representation enables more accurate node identification in natural environments, thereby supporting the development of intelligent and precise sugarcane harvesting equipment.

### 3.3 Model quantization acceleration

**Figure 4:**
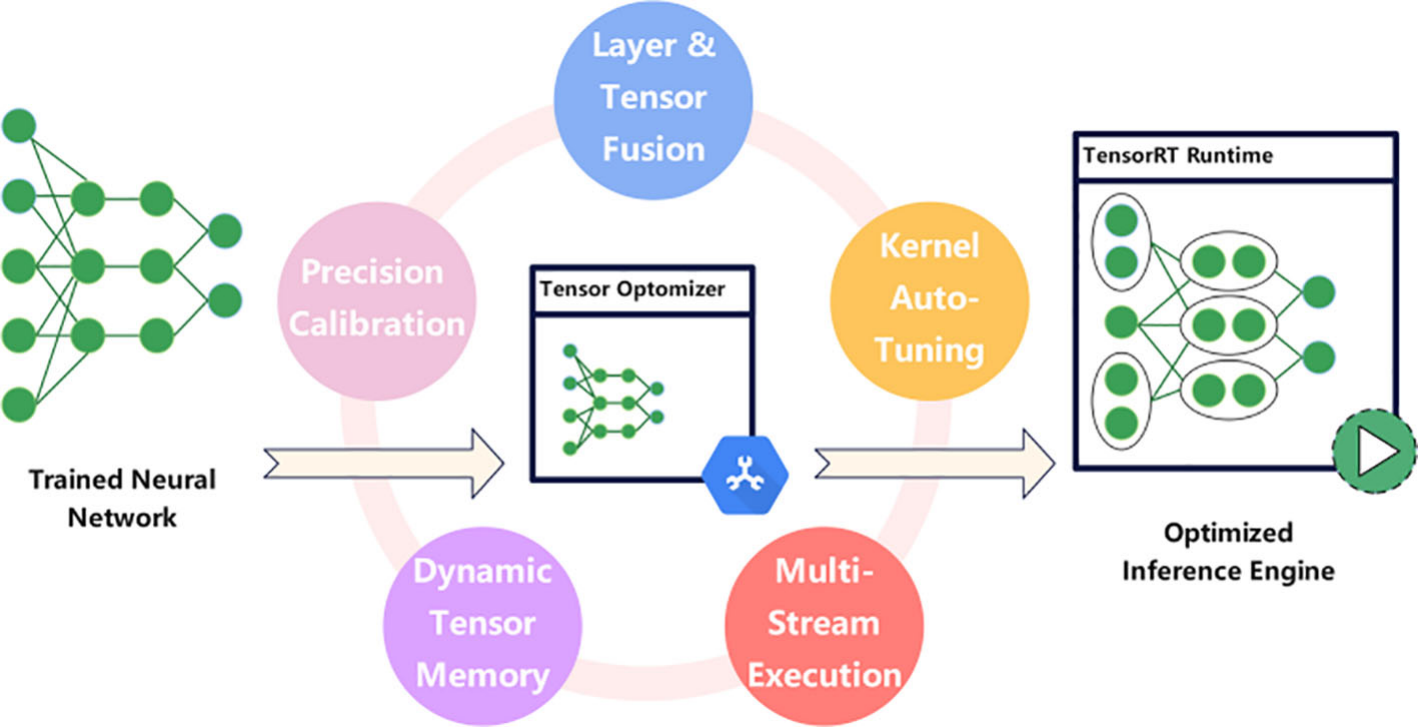
TensorRT architecture diagram.

This study employs a quantization acceleration approach to optimize the performance of the sugarcane intelligent recognition and harvesting model. Given the relatively fixed characteristics of sugarcane and the lower complexity of object recognition, as shown in **Figure 4**, the model is quantized using NVIDIA’s TensorRT platform through weight and activation precision calibration. This maximizes throughput and significantly boosts inference speed while maintaining high accuracy. Furthermore, the combination of layers and tensors optimizes GPU memory and bandwidth utilization efficiency. The automatic kernel tuning mechanism allows the selection of optimal data layers and algorithms based on the target GPU platform, thereby achieving further performance improvements. Simultaneously, a dynamic tensor memory management strategy effectively minimizes memory footprint and reuses tensor memory, ensuring efficient resource utilization. Finally, a multi-stream execution mechanism is designed to support parallel processing of multiple input streams. These optimization measures reduce the performance requirements of edge devices in planting areas, reduce actual deployment costs, and facilitate the realization of a real-time and efficient sugarcane node recognition system.

In summary, the TensorRT-based quantization and acceleration strategy significantly reduce the model’s inference time and memory footprint, enabling real-time detection on low-power edge devices such as NVIDIA Orin NX. These enhancements lower the operational costs and energy requirements in practical sugarcane farming environments, making intelligent node recognition more accessible and scalable for small- and medium-sized farms.

## 4 Experimental results and analysis

### 4.1 Experimental environment and parameters

To fairly evaluate the performance of each algorithm, all algorithms were ensured to use the same training platform and hyperparameter settings in the experiment. The following are the details of the experimental platform used in this study: The processor adopts Intel Xeon Gold 62656 processor, the main frequency is 3.60 GHz, equipped with 48 physical cores and 24 threads, and the memory capacity is 1024 GB. The graphics card uses NVIDIA RTX 4090 with 24 GB of video memory. The operating system is Ubuntu 18.04. CUDA 11.8.130, CUDNN 8.6.0, NVIDIA driver 535.104, OpenCV 4.8.0, and training framework PyTorch 2.0.1 are installed in the system.

1. Parameter Settings: This study selects the officially provided pre-trained models YOLOv5l and YOLOv8l as the basic models. The dataset image size is set to 640 × 640 pixels to reduce the computational burden and maintain sufficient details, thereby improving training efficiency. The experiment implemented 10,000 rounds of training and testing until the results stabilized. The batch size is set according to the maximum network performance to maximize the use of computing resources and ensure that each iteration can reasonably process samples. In addition, the initial learning rate is set to 0.01, and the momentum is set to 0.90 to accelerate model convergence and suppress oscillations during gradient descent, ensuring the smoothness and efficiency of the training process. Weight decay is set to 0.0005, which aims to regularize and prevent the model from overfitting.
2. Training Strategy: In order to optimize the training process, the K-Means clustering algorithm is used to accurately determine the optimal aspect ratio of the anchor boxes, and automatically identify the optimal cluster center to guide model training. In order to enhance the generalization ability and robustness of the model, the experiment adopts a variety of image enhancement techniques, including: using Mosaic technology to enhance sample diversity and background complexity; applying Mixup technology to generate new samples through linear combination of images and labels; introducing EMA technology to smooth model parameters and enhance stability; in terms of color space, adjusting the HSV color space to enhance the saturation and brightness of images to adapt to different lighting and color changes; in addition, Flip technology is used for horizontal flipping to improve the model’s ability to recognize symmetry. In addition, an EarlyStopping mechanism was adopted to monitor validation performance during training and automatically halt optimization when no improvement was observed after a set number of epochs, thereby effectively preventing overfitting and ensuring generalization.

### 4.2 Evaluation metrics

In this study, to more effectively evaluate the performance of the sugarcane detection model, we selected Precision, Recall, mean Average Precision at 0.5 IoU threshold (mAP@0.5), F1-score, inference time, and model size as key metrics. The calculation formulas are detailed below as Equations 1 to 4.


(1) $$P=\frac{T_{P}}{T_{P}+F_{P}}$$

(2) $$R=\frac{T_{P}}{T_{P}+F_{N}}$$

(3) $$mAP=\frac{\sum_{i=1}^{C}AP_{i}}{C}$$

(4) $$F_{1}=\frac{2PR}{P+R}$$

Within this framework, True Positives (TP) refer to samples with actual positive labels and positive prediction results; False Positives (FP) refer to samples with actual negative labels but positive prediction results; and False Negatives (FN) are samples that are actually positive but predicted as negative, representing unidentified sugarcane. These three are the basis for the calculation of precision and recall, which in turn affect the calculation of mAP and F1-score. Precision, Recall, mAP, and F1-score are important metrics for measuring the performance of a detection model. Among them, mAP represents the area under the Precision-Recall curve, reflecting the overall effectiveness of the model. Inference time represents the time required to detect a single sugarcane image, assessing the efficiency of the model, which is particularly important in practical applications. Model size refers to the storage space occupied by the model. These metrics collectively constitute a comprehensive evaluation of the performance of the developed model.

### 4.3 Comparison experiments with different advanced recognition methods

To verify the effectiveness of the proposed EdgeSugarcane model, we conducted comparative experiments with several mainstream detection models, including YOLOv5, YOLOv8, and multiple improved YOLOv8 variants (YOLOv8-EfficientVit, YOLOv8-c2f-Cloatt, YOLOv8-faster_ema, and YOLOv8-bifpn). The evaluation metrics included precision, recall, mAP, inference time, and model size, with a particular focus on balancing detection accuracy and real-time performance in practical applications.

As shown in **Table 2**, although YOLOv5 demonstrated a slightly smaller model size and faster baseline inference time than YOLOv8, it also exhibited lower recall and mAP. Considering the need to detect small and partially occluded sugarcane nodes under complex lighting and environmental conditions, we selected YOLOv8 as the base framework due to its architectural advantages—namely, a decoupled detection head, improved multi-scale feature fusion, and better semantic representation capabilities. Additionally, YOLOv8 provides stronger compatibility with deployment frameworks such as TensorRT, making it more suitable for optimization on edge hardware. Building upon YOLOv8, the proposed EdgeSugarcane model introduces a Triplet Attention module and applies FP16-based quantization using TensorRT. As a result, EdgeSugarcane achieves a precision of 0.935, a recall of 0.800, and an mAP of 0.870, with a single-frame inference time of only 9.3 ms and a model size of 87.6 MB. Compared to the original YOLOv8, it improves mAP by 0.6%, F1-score by 2.0%, precision by 2.9%, and recall by 1.4%, while reducing inference latency by 44%. It is also 28% faster than YOLOv5, and 36.7%–48.3% faster than other YOLOv8 variants. These results confirm that our improvements deliver a highly accurate, efficient, and deployable sugarcane node detection model, especially well-suited for real-time operation on resource-constrained agricultural edge devices.

**Table 2:** Comparison of detection performance of different networks.

Evaluation Metrics model	P	R	F1	mAP	Inference time	Model size
Yolov5	0.910	0.770	0.834	0.840	12.9	56.2
Yolov8	0.906	0.786	0.842	0.864	16.6	87.6
Yolov8-EfficientVit	0.933	0.774	0.846	0.801	15.6	8.7
Yolov8-c2f-Cloatt	0.911	0.794	0.848	0.806	16.9	89.0
Yolov8-faster ema	0.899	0.802	0.848	0.845	18.0	52.2
Yolov8-bifpn	0.884	0.810	0.846	0.856	14.7	63.0
EdgeSugarcane	0.935	0.800	0.862	0.870	9.30	87.6

**Figure 5:**
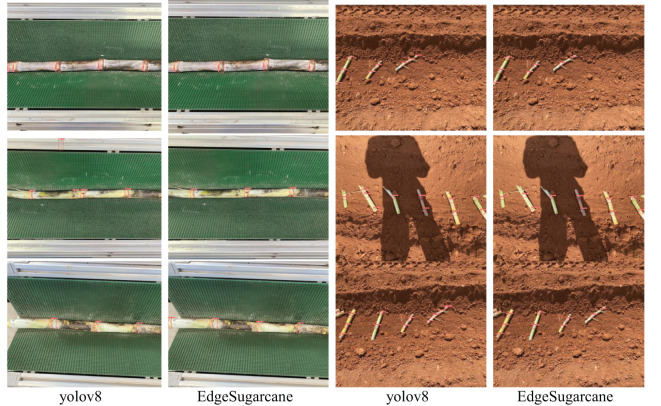
Comparison of detection effects in different environments.

**Figure 5** compares the detection performance of the improved EdgeSugarcane model and YOLOv8 in indoor sugarcane cutter and outdoor planter operating environments. The results show that the improved model has better recognition accuracy than YOLOv8, can more accurately identify sugarcane nodes, and significantly reduces false positives, false negatives, and the number of redundant bounding boxes. In the indoor sugarcane cutter operating scenario, YOLOv8 is prone to overlapping detections, incorrect judgments, and missed detections due to blurred edges of the nodes, resulting in a decrease in recognition accuracy. In contrast, EdgeSugarcane enhances feature discriminability through optimized algorithms and the Triplet Attention module, solving the problems of repeated detection, false positives, and missed detections of YOLOv8 in complex agricultural scenarios. In the outdoor planter operating scenario, YOLOv8 often has difficulty in correct recognition due to complex lighting conditions, while EdgeSugarcane can still maintain high recognition performance under these conditions. Thus, EdgeSugarcane demonstrates stronger adaptability and robustness, and can achieve accurate sugarcane node detection in diverse scenarios, providing high-precision, low-latency detection support for mechanized sugarcane operations.

### 4.4 Comparative experiments of different quantization methods

To reduce model size, decrease computational resource consumption, and improve inference speed while maintaining model performance, this section presents comparative experiments between the original EdgeSugarcane model and two quantization methods (TensorRT-FP16 and TensorRT-INT8). This is done to verify the optimization effects of quantization techniques in real-world deployments. The experiment investigates the impact of different quantization strategies on the performance of models in practical applications. **Table 3** presents the results of each model’s performance metrics.

**Table 3:** Analysis of quantization method comparison experiments.

Evaluation Metrics data	Original model (EdgeSugarcane)	TensorRT-FP16	TensorRT-INT8
P	0.935	0.935	0.399
R	0.8	0.8	0.167
mAP	0.87	0.87	0.258
Model Size (MB)	87.6	89.9	47.1
Inference Time (ms)	6.3	1.9	1.8
Preprocessing Time (ms)	0.3	0.9	0.7
Postprocessing Time (ms)	2.7	4.4	4.8

The TensorRT-FP16 deployment solution proposed in this study achieves a synergistic optimization of accuracy and efficiency in the task of sugarcane node detection. Experimental data demonstrates that while maintaining the original model’s lossless detection accuracy, the inference speed is significantly increased to 3.3 times that of the original model, and the model size remains virtually unchanged. Compared to INT8 quantization which suffers from accuracy degradation rendering it impractical, FP16 quantization maintains model robustness without requiring quantization-aware training, significantly reducing the complexity of heterogeneous computing platform adaptation. With these advantages, TensorRT-FP16 technology can effectively improve the performance of sugarcane node detection systems. It has millisecond-level detection speeds, meeting the needs of real-time field detection, and can be adapted to mobile devices. In scenarios with complex overlapping of nodes, it maintains high localization accuracy, reducing missed detections and false positives. Furthermore, the method is directly compatible with low-cost edge computing devices, facilitating the rapid deployment and application of node detection systems in the field.

### 4.5 Field experiments

In real-world orchard scenarios, cloud-based sugarcane recognition models struggle to meet real-time requirements due to limited computing resources on edge devices and unstable network conditions. To achieve end-to-end sugarcane recognition and localization, this study deploys the optimized EdgeSugarcane model to an NVIDIA Jetson Orin NX (16 GB, 1024-core Ampere GPU) edge computing device and systematically evaluates its performance in real orchard environments. The software environment used during deployment includes TensorRT version 8.5.2.2, CUDA 11.4.315, cuDNN 8.6.0.166, Ultralytics YOLOv8 version v8.1.0, and Python 3.8.10.

Experimental results in **Table 4** show that after applying TensorRT-FP16 optimization, the inference time on the Orin platform was reduced from 3.2 ms to 1.5 ms, achieving a 53.1% speed improvement. The total end-to-end processing time stabilized at 66.5 ms, with preprocessing and postprocessing times reduced by 62.5% and 58.1%, respectively. GPU memory usage decreased from 1.5 GB to 1.4 GB, reducing the overall memory footprint on the edge device. Across all configurations, the model maintained consistent performance, with a precision of 0.935, recall of 0.8, and mAP of 0.87, even in complex orchard conditions with overlapping nodes. These results confirm that FP16 quantization preserves the model’s representational capability while improving latency and resource efficiency. EdgeSugarcane demonstrates enhanced adaptability and robustness, supporting accurate node detection in varied scenarios and enabling high-precision, low-latency performance for mechanized sugarcane operations.

**Table 4:** Performance comparison of edgesugarcane model under different hardware platforms and optimization strategies.

Evaluation metricsData	EdgeSugarcane-TensorRT-FP16 on 4090	EdgeSugarcane on ORIN	EdgeSugarcane-TensorRT-FP16 on ORIN
P	0.935	0.935	0.935
R	0.8	0.8	0.8
mAP	0.87	0.87	0.87
Inference Time (ms)	1.9	3.2	1.5
Preprocessing Time (ms)	0.9	161.4	60.6
Postprocessing Time (ms)	4.4	10.5	4.4
Gpu memory	990mb	1.5g	1.4g

## 5 Discussion

This paper proposes a lightweight sugarcane node detection method called EdgeSugarcane, designed to address the challenges of sugarcane node identification in complex natural environments. By introducing an interactive attention mechanism across channel and spatial dimensions to improve the YOLOv8 network architecture, we enhanced the capability to extract node features. Furthermore, by combining the FP16 quantization technology of the TensorRT framework, we completed end-to-end deployment on the NVIDIA Orin NX edge device, achieving a balance between high accuracy and low resource consumption, and significantly improving the system’s real-time performance and environmental adaptability

In the field of sugarcane node detection, existing methods, such as those by Tan et al. (2014), Huang et al. (2017), Chen et al. (2021c), and Meng et al. (2019), primarily rely on manual feature extraction and machine learning algorithms. While these methods have achieved certain recognition rates in specific scenarios, they generally suffer from insufficient real-time performance and weak environmental robustness. In recent years, methods based on deep learning techniques, such as those by Dai et al. (2024b), Wang et al. (2022), and Xie et al. (2023), have significantly improved detection efficiency, but the model performance is still susceptible to environmental factors. In contrast, our proposed EdgeSugarcane method not only improves detection accuracy in complex environments but also significantly reduces single-frame inference time and end-to-end processing time, enabling it to meet the needs of applications requiring higher real-time performance.

**Figure 6:**
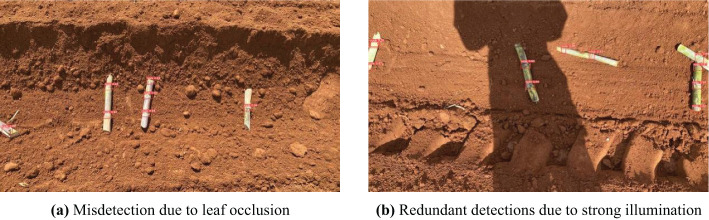
Detection failure analysis: **(a)** misdetection caused by leaf occlusion; **(b)** redundant detection under strong illumination.

The core innovation of this research lies in achieving efficient synergy between a lightweight detection model and edge computing, validating the field applicability of sugarcane node detection on the NVIDIA Orin NX device. Compared to traditional cloud-based approaches, this method significantly reduces computational resource consumption and latency while maintaining detection accuracy, enabling low-cost, high-efficiency sugarcane node detection. Although EdgeSugarcane performs excellently in most scenarios, it exhibits certain limitations in extreme environmental conditions, as shown in **Figure 6**. First, when dense sugarcane leaves heavily cover the node regions, the similarity between leaf textures and node features can cause the model to misidentify leaf edges as node boundaries. Second, under strong midday sunlight, glare on the node surface can create bright spots, leading the model to generate multiple overlapping bounding boxes on the same node. Future work will focus on addressing these issues and further optimizing the algorithm to promote the practial application of intelligent sugarcane cutting and planting equipment.

## 6 Conclusions

This paper presents EdgeSugarcane, a lightweight sugarcane node detection method based on an improved YOLOv8, which has been successfully deployed on the NVIDIA Orin NX edge computing device.

1. This paper proposes the EdgeSugarcane model, which enhances multi-scale feature representation by embedding a Triplet Attention module, effectively suppressing complex background interference. Experimental results demonstrate that EdgeSugarcane achieves a precision, recall, and mAP50 of 0.935, 0.8, and 0.87, respectively, on the test set. The mAP is improved by 0.6% compared to YOLOv8, the inference speed is increased by 44%, and the model size is 89.9MB. Compared with other methods, EdgeSugarcane significantly improves the accuracy and efficiency of sugarcane node identification.
2. An acceleration-optimized deployment strategy based on TensorRT is proposed, achieving synergistic optimization of accuracy and efficiency. TensorRT-FP16 significantly increases the inference speed to 1.9ms under lossless accuracy conditions, achieving 3.3 times that of the original model. At the same time, the model size remains virtually unchanged. The millisecond-level detection speed meets the real-time requirements of field applications, high accuracy ensures localization in complex scenarios, and it is easy to deploy on low-power agricultural hardware, reducing application costs.
3. EdgeSugarcane was deployed on the NVIDIA Orin NX edge device, achieving end-to-end application of the sugarcane node detection algorithm, and robustness verification was performed in field environments. On the NVIDIA Orin NX device, the single-frame inference time, pre-processing time, and post-processing time were 1.5ms, 60.6ms, and 4.4ms, respectively. The edge deployment scheme effectively avoids network latency issues, and maintains an mAP value of 87% in complex field scenarios such as strong light and shadows, verifying its robustness in complex environments. This proves the feasibility of the method proposed in this paper and provides an important technical reference for intelligent sugarcane cutting and planting localization.

## Data Availability

The raw data supporting the conclusions of this article will be made available by the authors, without undue reservation.
